# An age-and sex-matched postoperative therapy should be required in thyroid papillary carcinoma

**DOI:** 10.3389/fendo.2024.1339191

**Published:** 2024-06-21

**Authors:** Caigu Yan, Jinjin Sun, Xianghui He, Lanning Jia

**Affiliations:** ^1^ Department of General Surgery, The Second Hospital of Tianjin Medical University, Tianjin, China; ^2^ Department of General Surgery, Tianjin Medical University General Hospital, Tianjin, China

**Keywords:** papillary thyroid carcinoma, lymph node metastasis, TSH, ^131^I, SLC5A5

## Abstract

**Background and purpose:**

Thyroid papillary carcinoma (PTC) had a high possibility of recurrence after surgery, and thyroid stimulating hormone (TSH) suppression and radioactive iodine (^131^I) were used for postoperative therapy. This study explored the potential mechanism of lymph node metastasis (LNM) and aimed to develop differentiated treatments for PTC.

**Method:**

This study explored the risk factors of lymph node metastasis in PTC by analyzing the clinical information of 2073 cases. The Cancer Genome Atlas Thyroid Cancer (TCGA-THCA) and the Gene Expression Omnibus (GEO) databases of gene expression were analyzed to identify the interrelationships between gene expression to phenotype.

**Results:**

Analyzing clinical data, we found that male gender, younger age, larger tumor size, and extra-thyroidal extension (ETE) were risk significant risk factors for lymph node metastasis(P<0.05). Conversely, thyroid function parameters such as TSH, FT3, FT4, TSH/FT3, and TSH/FT4 didn’t correlate with LNM(P>0.05), and TSH levels were observed to be higher in females(P<0.05). Gene expression analysis revealed that SLC5A5 was down-regulated in males, younger individuals, and those with lymph node metastasis, and a lower level of SLC5A5 was associated with a worse disease-free survival(P<0.05). Additionally, our examination of single-cell RNA sequencing (scRNA-seq) data indicated that SLC5A5 expression was reduced in tumors and lymph node metastasis samples, correlating positively with the expression of TSHR.

**Conclusion:**

The impact of TSH on PTC behavior remained unclear, while the capacity for absorbing ^131^I in dependence on SLC5A5 showed variations across different genders and ages. We conclude that postoperative treatment of PTC should take into account the differences caused by gender and age.

## 1 Background

Papillary thyroid carcinoma (PTC) is one of the most frequently diagnosed malignancies of the endocrine system worldwide (1). Surgery is the preferred treatment for PTC, which has a satisfactory prognosis in an early stage (2). However, studies have revealed that the recurrence rate of PTC within 5 years is a high level, ranging from 2%-13% (3–6), which was difficult in the treatment of PTC. To address this issue, the use of thyroid-stimulating hormone (TSH) suppression has been advocated for postoperative patients to mitigate the growth of tumor cells and reduce the risk of recurrence (7, 8). In addition, the American thyroid association 2016 guidelines recommend radioactive iodine (131I) for patients with a high risk of recurrence, such as those with residual disease or metastasis (7, 9–11). However, patients exposed to a low level of TSH are at risk of developing subclinical hyperthyroidism including cardiovascular disease. For menopausal women, excessive TSH suppression may lead to osteoporosis and increase the risk of fractures (12–15). Patients receiving ^131^I therapy may experience damage to the parotid gland and an increased risk of secondary tumors, which increases with the radiation dose (16, 17). Currently, decisions regarding the extent of TSH suppression and the dosage of ^131^I are primarily based on pathological invasion assessments, which vary among physicians and surgeons. Some clinicians favor more aggressive treatments to prevent recurrences (18, 19).

Some studies have indicated that PTC exhibits varying biological behaviors across different age groups and gender demographics (20, 21). Specifically, women were considered to be more prone to developing PTC, yet they typically experienced milder behavior and a more favorable prognosis, particularly among pre-menopausal females. In addition, younger patients display a more positive prognosis but are at an increased risk of recurrence (22, 23). A study on active observation of papillary thyroid microcarcinoma(PTMC) reported that tumors are more likely to progress in younger patients (24). The diverse hormonal and immunological internal environments influenced by gender and age differences could be responsible for these variable behaviors.

Our study integrated the clinical data, preoperative examinations, and postoperative pathology of the PTC patients to delineate the clinical trends within diverse subgroups. Through analyses of the gene expression database, we investigated the gene expression levels across various subgroups to unravel the mechanism underpinning the observed phenotype. This research aimed to provide a more personalized and less harmful postoperative therapeutic regimen.

## 2 Materials and methods

### 2.1 Clinical data

**Figure 1:**
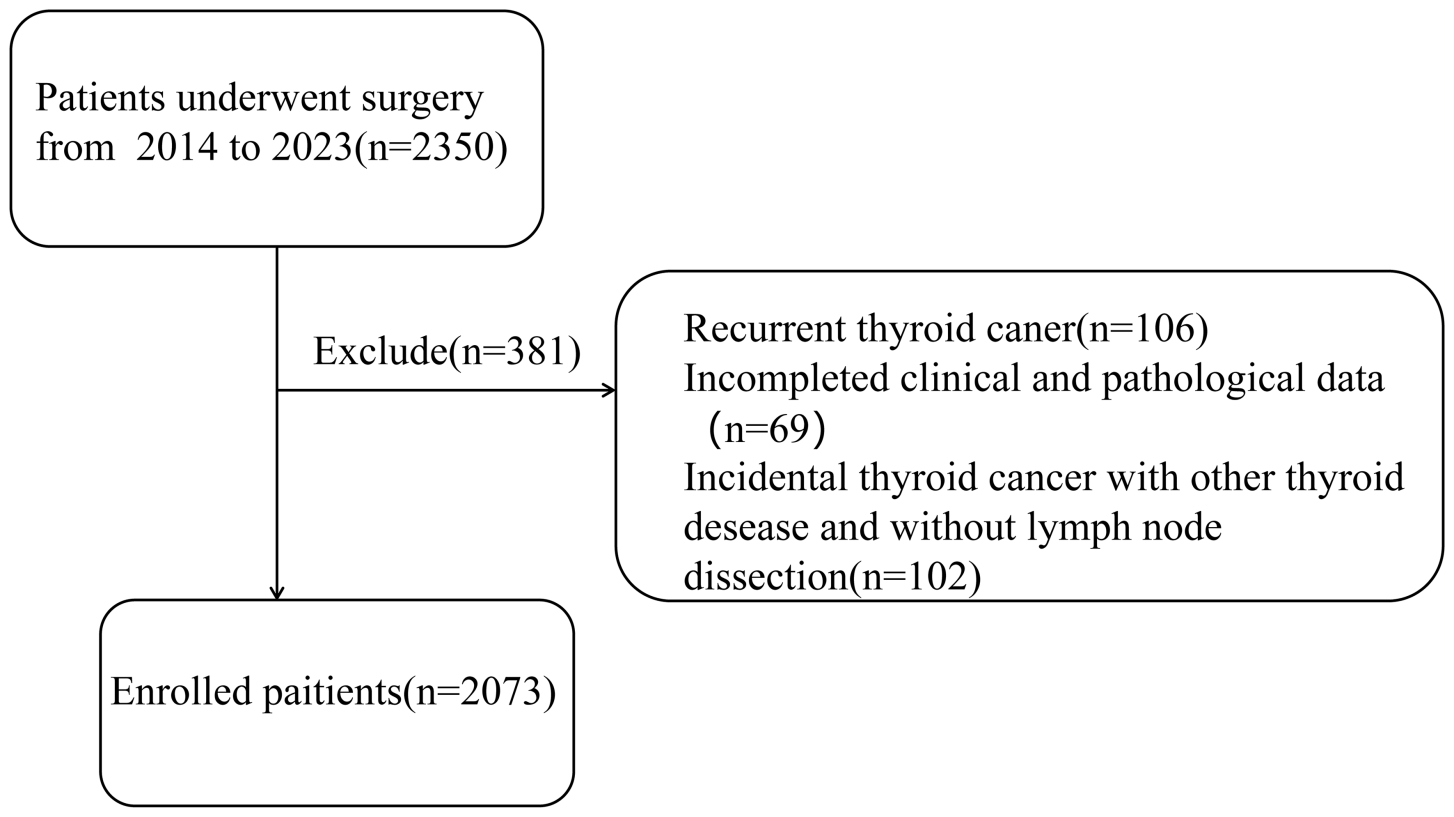
The flow chart of the patients enrolled in this study.

A total of 2073 patients from 2014 to 2023 were selected in this study, and all of them were diagnosed with PTC and underwent surgery by the same doctor. What’s more, in our center, all patients diagnosed with PTC would undergo routine prophylactic central compartment lymph node dissection, and the pathological results were obtained separately by two experienced pathologists. This study passed the ethical review based on the Declaration of Helsinki and obtained informed consent from the patients. Inclusion criteria were: 1) initial thyroid cancer surgery; 2) completed clinical and pathological data; Exclusion criteria were: 1) recurrent thyroid cancer; 2) incidental thyroid cancer without central lymph node dissection (**Figure 1**).

We set 45 years old as the cutoff age, which was aligned with the mean age of our entire cohort and conformed to the changes in female hormone levels. Based on the character of clinical data, all participants were divided as follows: LNM (absent and present), tumor size(less than 1 cm or more than 1 cm), extrathyroidal extension (limited in capsule or violating surrounding tissues), status of thyroid function (hypothyroidism(TSH>4.94uIU/L or FT3 ≤ 2.43pmol/L or FT4 ≤ 9.01pmol/L), normal thyroid function (TSH:0.35uIU/L-4.94uIU/L, FT3:2.43pmol/L-6.01pmol/L and FT4:9.01pmol/L-19.05) and hyperthyroidism (TSH<0.35uIU/L or FT3>6.01pmol/L or FT4>19.05pmol/L), and the level of TSH(high: TSH > 2uIU/L, low: TSH ≤ 2uIU/L). What’s more, the ratio of TSH/FT3 and TSH/FT4 were used to represent the response of the hypothalamic-pituitary-thyroid axis.

### 2.2 Public data of gene expression

We downloaded the bulk gene expression data and clinical data of PTC from the TCGA database UCSC (https://xenabrowser.net/) and deleted the cases without LNM information. Further, 497 cases with complete age and sex information were selected, and the included genes were normally detected in at least 75% of participants. scRNA-seq data were extracted from the Gene Expression Omnibus (GEO) dataset (GSE184362), and the quality control was performed according to the standard Seurat process.

### 2.3 Statistical analysis

The SPSS 22.0 software was used to analyze the statistical data. The continuous measurement data were expressed as $\bar{\text{x}}$ ± S and a t-test was performed for statistical analysis. What’s more, categorical variables such as sex, age, tumor size, degree of extrathyroidal extension, and status of thyroid function were counted by their frequency, and binary logistic regression analysis was performed for statistical analysis. Then, multivariate analysis was performed by multivariate logistic regression analysis. A P value <0.05 indicated a statistically significant difference. The different expressed genes (DEGs) from the TCGA database were screened using the “DESeq2” package in R 4.2.2 software. The DEGs were defined as the average gene expression ratio and two times the standard deviation, with P <0.05.

## 3 Results

### 3.1 Clinical data and single-factor analysis of LNM

According to the characteristics of clinical data, the age range was 13 to 89 years, and with 1060 patients under 45 years old,1013 were older than 45 years. All patients were divided into 556 males and 1517 females, and the ratio of males to females was 1:2.73. The average age of males was 44.2, and 45.3 for females. Among them, 942 were PTMC with a diameter less than 1cm, and 1131 carcinomas with a diameter larger than 1cm. In 1169 cases, the tumor was limited to the capsule, but in 454 cases invaded surrounding tissues. According to the definition of thyroid function, there were 121 with hypothyroidism, 1826 with normal thyroid function, and 126 patients with hyperthyroidism. Among all patients, 1101(53.1%) cases occurred lymph node metastasis, and 972(46.9%) without. The univariate analysis performed by binary logistic regression analysis showed the younger age, male gender, larger tumor size, and ETE were risk factors for LNM (P<0.05), but the factors of thyroid function weren’t(P>0.05) (**Table 1**). Then, We showed interest in the levels of FT3, FT4, TSH, TSH/FT3, and TSH/FT4 in different lymph node metastatic status, but the results of the t-test showed no significant difference here (P>0.05) (**Table 2**). What’s more, we conducted analyses to compare the thyroid function and the levels of FT3, FT4, TSH, TSH, TSH/FT3, and TSH/FT4 in different statuses of age, sex, tumor size, and capsule invasion (**Table 3**). The study’s findings revealed no significant differences in thyroid function status or the levels of FT3, FT4, TSH, TSH, TSH/FT3, and TSH/FT4 when comparing different age groups and varying degrees of capsule invasion(P>0.05). However, upon conducting t-tests and binary logistic regression analyses, it was observed that within subgroups of larger sizes, there was a statistically significant increase in the levels of FT3 and FT4 (P<0.05), whereas the levels of TSH, TSH/FT3, and TSH/FT4 did not exhibit any significant differences (P>0.05). Additionally, gender-based comparisons indicated notable variations in thyroid function, as well as the levels of FT3, FT4, TSH, TSH/FT3, and TSH/FT4, between females and males (P<0.05). Women presented with a higher prevalence of hypothyroidism; the mean TSH level among women was 2.54 uIU/L, compared to 2.11 uIU/L in men. Moreover, the mean TSH/FT3 ratio was significantly higher in women at 0.61, contrasting with 0.46 in men.

**Table 1:** Clinicopathological characteristics and univariate analysis of the 2073 PTC patients.

Parameter	LNM(+) (1101)	LNM(-) (972)	OR (95% CI)	P
Gender			1.733(1.420–2.115)	0.000
Female	751(68.2%)	766(78.8%)		
Male	350(31.8%)	206(21.2%)		
Tumor size (cm)			2.043(1.713–2.435)	0.000
≤1	410(37.2%)	532(54.7%)		
>1	691(62.8%)	440(45.3%)		
Age			0.566(0.475–0.673)	0.000
<45 years	636(57.8%)	424(43.6%)		
≥45 years	465(42.2%)	548(56.4%)		
ETE			1.615(1.304–1.999)	0.000
Limiting in capsule	819(74.4%)	801(82.4%)		
Violating surrounding tissues	282(25.6%)	171(17.6%)		
Thyroid function				0.834
Hypothyroidism	63(5.7%)	58(6.0%)		
Normal	974(88.4%)	852(87.7%)	1.052(0.728–1.521)	0.786
Hyperthyroidism	64(5.8%)	62(6.3%)	0.950(0.577–1.566)	0.841

PTC, papillary thyroid carcinoma; ETE, extra thyroidal extension; LNM, lymph node metastasis; OR, Odds Ratio.

**Table 2:** Clinicopathological characteristics and t-test of the 2073 PTC patients.

Parameter	LNM(+) (1101)	LNM(-) (972)	Deviation (95% CI)	P
FT3	4.55 ± 1.42	4.54 ± 0.98	-0.005(-0.112–0.101)	0.926
FT4	13.22 ± 2.37	13.20 ± 2.21	-0.013(-0.212–0.185)	0.898
TSH	2.49 ± 4.73	2.35 ± 2.32	-0.133(-0.461–0.194)	0.423
TSH/FT3	0.61 ± 1.74	0.53 ± 0.53	-0.074(-0.188–0.039)	0.199
TSH/FT4	0.21 ± 0.60	0.20 ± 0.33	-0.007(-0.050–0.035)	0.729

PTC, papillary thyroid carcinoma; LNM, lymph node metastasis.

**Table 3:** Univariate analysis of risk factors to different age and gender subgroups.

Parameter	<45 years (n=1060)	≥45 years (n=1013)	P	Male (n=556)	Female (n=1517)	P
Thyroid function			0.138			0.009
Low*	52(4.9%)	69(6.8%)		18(3.3%)	103(6.8%)	
Normal	947(89.3%)	879(86.8%)	0.059	500(89.9%)	1326(87.4%)	0.003
High**	61(5.8%)	65(6.4%)	0.391	38(6.8%)	88(5.8%)	0.005
FT3(pmol/L)	4.55 ± 0.82	4.54 ± 1.55	0.860	4.45 ± 0.92	4.81 ± 1.81	0.000
FT4(pmol/L)	13.23 ± 2.17	13.19 ± 2.43	0.630	13.10 ± 2.33	13.51 ± 2.18	0.000
TSH(uIU/L)	2.45 ± 4.74	2.39 ± 2.46	0.743	2.54 ± 4.04	2.11 ± 3.01	0.022
TSH/FT3	0.59 ± 1.74	0.55 ± 0.61	0.515	0.61 ± 1.47	0.46 ± 0.73	0.024
TSH/FT4	0.21 ± 0.61	0.20 ± 0.32	0.868	0.22 ± 0.55	0.17 ± 0.27	0.039
Parameter	<1cm (n=942)	≥1cm (n=1131)	P	Limiting in capsule (n=1620)	Tissue invading (n=453)	P
Thyroid function			0.005			0.752
Low*	51(5.4%)	70(6.2%)		97(6.0%)	24(5.3%)	
Normal	851(90.3%)	975(86.2%)	0.342	1427(88.1%)	399(88.1%)	0.603
High**	40(4.3%)	86(7.6%)	0.091	96(5.9%)	30(6.6%)	0.450
FT3(pmol/L)	4.48 ± 0.75	4.60 ± 1.52	0.018	4.56 ± 1.33	4.50 ± 0.76	0.429
FT4(pmol/L)	13.07 ± 2.06	13.32 ± 2.47	0.014	13.23 ± 2.36	13.12 ± 2.07	0.381
TSH(uIU/L)	2.39 ± 3.83	2.45 ± 3.77	0.710	2.44 ± 4.16	2.34 ± 1.95	0.622
TSH/FT3	0.57 ± 1.40	0.57 ± 1.25	0.972	0.58 ± 1.46	0.54 ± 0.49	0.537
TSH/FT4	0.20 ± 0.58	0.20 ± 0.40	0.880	0.20 ± 0.52	0.36 ± 0.17	0.993

LNM, lymph node metastasis; PTC, papillary thyroid carcinoma; Low*, Hypothyroidism; High**, Hyperthyroidism.

We conducted a further investigation to elucidate the influence of thyroid function and the concentrations of FT3, FT4, TSH, TSH/FT3, and TSH/FT4 on lymph node metastasis (LNM) across various patient subgroups (**Tables 4**, **5**). Employing chi-square and t-test analyses within subgroups stratified by sex and age, our findings revealed no significant association between thyroid function and the levels of FT3, FT4, TSH, TSH/FT3, and TSH/FT4 with the incidence of LNM in these demographic subgroups (P>0.05). Additionally, acknowledging that larger tumor size and extrathyroidal extension are established risk factors for lymph node metastasis, we aimed to investigate the relevance of thyroid function parameters, including serum levels of FT3, FT4, TSH, TSH/FT3, and TSH/FT4, concerning LNM across various tumor sizes and invasive states, and the results indicated that these thyroid function-related factors did not exert a significant influence on the incidence of LNM (P > 0.05).

**Table 4:** Univariate analysis of risk factors to different age and gender subgroups.

Parameter	<45 years (n=1060)			≥45 years (n=1013)		
	LNM(+) (n=636)	LNM(-) (n=424)	P	LNM(+) (n=465)	LNM(-) (n=548)	P
Thyroid function			0.493			0.455
Low*	29(4.6%)	23(5.4%)		34(7.3%)	35(6.4%)	
Normal	574(90.3%)	373(88.0%)	0.488	400(86.0%)	479(87.4%)	0.366
High**	33(5.1%)	28(6.6%)	0.859	31(6.7%)	34(6.2%)	0.034
FT3(pmol/L)	4.52 ± 0.75	4.58 ± 0.91	0.270	4.57 ± 2.01	4.51 ± 1.03	0.510
FT4(pmol/L)	13.30 ± 2.22	13.14 ± 2.09	0.248	13.11 ± 2.56	13.25 ± 2.30	0.337
TSH(uIU/L)	2.48 ± 5.71	2.41 ± 2.68	0.809	2.49 ± 2.90	2.31 ± 2.00	0.229
TSH/FT3	0.62 ± 2.20	0.54 ± 0.60	0.446	0.58 ± 0.74	0.53 ± 0.47	0.135
TSH/FT4	0.21 ± 0.76	0.20 ± 0.28	0.736	0.20 ± 0.26	0.20 ± 0.36	0.952
Parameter	Male (n=556)			Female (n=1517)		
	LNM(+) (n=350)	LNM(-) (n=206)	P	LNM(+) (n=751)	LNM(-) (n=766)	P
Thyroid function			0.986			0.728
Low*	11(3.1%)	7(3.4%)		52(6.9%)	51(6.7%)	
Normal	315(90.0%)	185(89.8%)	0.871	659(87.7%)	667(87.1%)	0.878
High**	24(6.9%)	14(6.8%)	0.883	40(5.3%)	48(6.2%)	0.488
FT3(pmol/L)	4.85 ± 2.16	4.74 ± 0.94	0.505	4.41 ± 0.85	4.49 ± 0.98	0.086
FT4(pmol/L)	13.44 ± 2.09	13.6 ± 2.33	0.291	13.11 ± 2.49	13.09 ± 2.16	0.818
TSH(uIU/L)	2.13 ± 3.02	2.07 ± 3.00	0.832	2.65 ± 5.34	2.43 ± 2.09	0.278
TSH/FT3	0.47 ± 0.77	0.45 ± 0.66	0.755	0.67 ± 2.04	0.55 ± 0.49	0.126
TSH/FT4	0.17 ± 0.25	0.17 ± 0.31	0.992	0.23 ± 0.71	0.21 ± 0.33	0.538

LNM, lymph node metastasis; ETE, extrathyroidal extension; Low*, Hypothyroidism; High**, Hyperthyroidism.

**Table 5:** Univariate analysis of risk factors to different tumor size and ETE subgroups.

Parameter	<1cm (n=942)			≥1cm (n=1131)		
	LNM(+) (n=410)	LNM(-) (n=532)	P	LNM(+) (n=691)	LNM(-) (n=440)	P
Thyroid function			0.654			0.429
Low*	24(5.8%)	27(5.1%)		39(5.6%)	31(7.0%)	
Normal	371(90.5%)	480(90.2%)	0.628	603(87.3%)	372(84.6%)	0.310
High**	15(3.7%)	25(4.7%)	0.361	49(7.1%)	37(8.4%)	0.874
FT3	4.44 ± 0.73	4.51 ± 0.76	0.194	4.61 ± 1.70	4.59 ± 1.19	0.796
FT4	13.05 ± 2.05	13.09 ± 2.08	0.790	15.18 ± 2.54	16.28 ± 2.35	0.741
TSH	2.56 ± 5.47	2.27 ± 1.70	0.231	2.44 ± 4.23	2.46 ± 2.89	0.923
TSH/FT3	0.65 ± 2.06	0.51 ± 0.40	0.188	0.58 ± 1.51	0.55 ± 0.65	0.731
TSH/FT4	0.23 ± 0.86	0.18 ± 0.18	0.257	0.20 ± 0.37	0.22 ± 0.44	0.330
Parameter	Limiting in capsule (n=1620)			Tissue invading (n=453)		
	LNM(+) (n=819)	LNM(-) (n=801)	P	LNM(+) (n=282)	LNM(-) (n=171)	P
Thyroid function			0.923			0.809
Low*	48(5.9%)	49(6.1%)		15(5.3%)	9(5.3%)	
Normal	724(88.4%)	703(87.8%)	0.811	250(88.7%)	149(87.1%)	0.988
High**	47(5.7%)	49(6.1%)	0.942	17(6.0%)	13(7.6%)	0.665
FT3	4.57 ± 1.60	4.54 ± 1.00	0.604	4.47 ± 0.71	4.56 ± 0.84	0.249
FT4	14.81 ± 2.46	14.85 ± 2.24	0.985	13.18 ± 2.10	13.05 ± 2.04	0.533
TSH	2.52 ± 5.36	2.36 ± 2.41	0.449	2.38 ± 2.02	2.29 ± 1.83	0.617
TSH/FT3	0.62 ± 1.99	0.54 ± 0.56	0.231	0.56 ± 0.54	0.51 ± 0.38	0.326
TSH/FT4	0.21 ± 0.68	0.19 ± 0.27	0.499	0.19 ± 0.22	0.22 ± 0.51	0.395

LNM, lymph node metastasis; PTC, papillary thyroid carcinoma; ETE, extrathyroidal extension; Low*, Hypothyroidism; High**, Hyperthyroidism.

### 3.2 Multi-factor analysis and nomogram of LNM

**Figure 2:**
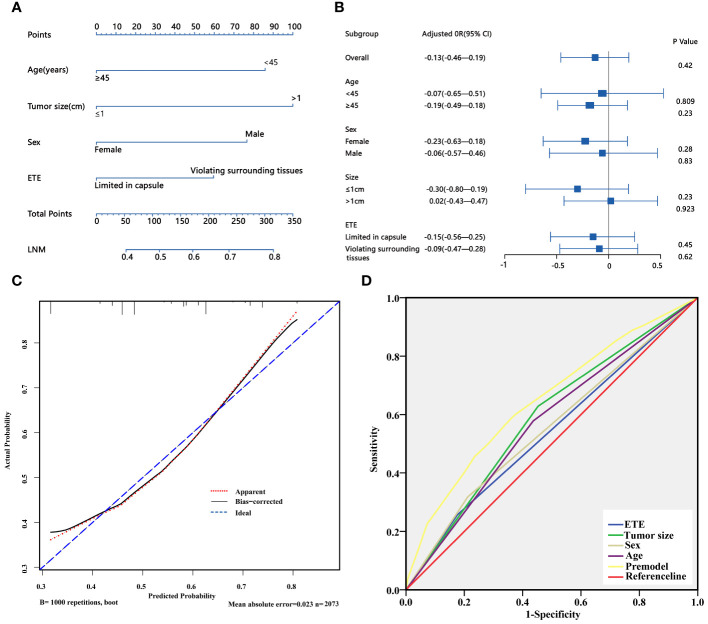
**(A)** Nomogram for predicting LNM in PTC patients; **(B)** The forest plot shows the impact of TSH on LNM in different subgroups. **(C)** Discrimination plot of the LNM predict mode. **(D)** ROC curve of the LNM predict model. LNM, lymph node metastasis; ETE, extrathyroidal extension.

Based on the above univariate analysis, factors that may have associations with LNM (P<0.05), including sex, age, tumor size, and degree of capsule invasion were included in the multivariate logistic regression model. The results showed that the larger size (OR=1.985, 95% CI 1.657–2.377; P<0.05), extrathyroidal extension (OR=1.505, 95% CI 1.206–1.877; P<0.05), male gender (OR=1.690, 95% CI 1.377–2.075; P<0.05) and younger age (OR=1.802, 95% CI 1.506–2.156; P<0.05) were independent risk factors for LNM (**Table 6**). Based on the results of multivariate logistic regression analysis, a nomogram predictive model was created to show the weight of each factor in this model (**Figure 2A**). To explore the impact of TSH on lymph node metastasis in each subgroup, a forest map was drawn, and the results showed that TSH levels had no significant effect on each subgroup(P>0.05) (**Figure 2B**). After 1000 internal verifications, an internal alignment curve was plotted and showed the average absolute error of the actual risk probability and the predicted risk probability of the model was 0.023 (P<0.05) (**Figure 2C**). The area under the receiver operating characteristic(ROC) curve was 0.647 (95% CI 0.624–0.671; P<0.05), indicating that the diagnostic efficiency of this model was superior to that of a single factor model (**Figure 2D**).

**Table 6:** Predictive factors of LNM in PTC patients in multiple logistic regression analysis.

Parameter	p	Adjusted OR	95%CI for Adjusted OR	
			Lower	Upper
Sex	0.000	1.690	1.377	2.075
Age	0.000	1.802	1.506	2.156
Tumor size	0.000	1.985	1.657	2.377
ETE	0.000	1.505	1.206	1.877
Constants	0.000	0.463	——	——

LNM, lymph nodes metastasis; PTC, papillary thyroid carcinoma; ETE, extrathyroidal extension.

### 3.3 TCGA bulk gene expression and DEGs

**Figure 3:**
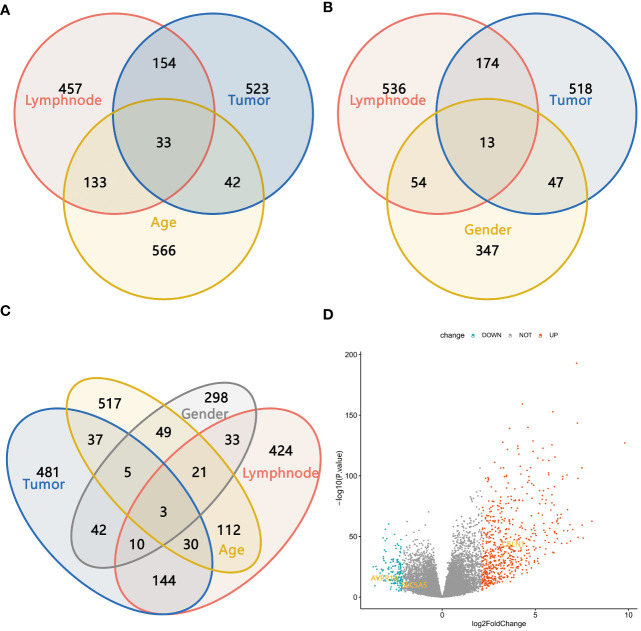
**(A–C)** The Venn maps based on the intersection DEGs of different subgroups; **(D)** Three intersection genes with the same trend in four subgroups.

**Figure 4:**
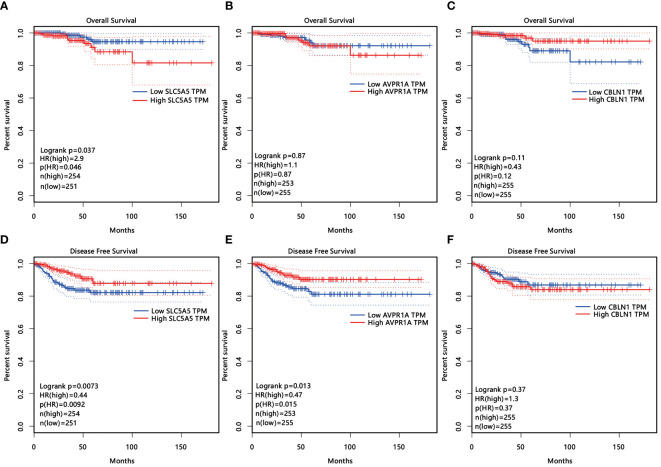
**(A–F)** The influence of three age- and sex-related DEGs (SLC5A5, AVPR1A, CLNB1) on overall survival **(A–C)** and disease-free survival **(D-F)**.

Among the 497 samples, 444 were tumor tissues and 53 were para-tumor tissues. All participants were divided into subgroups according to the tissue sources, LNM status, sex, and age. The baseline was females, and older age, which conformed to the status of LNM. Genes were defined as upregulated, downregulated, and unchanged in different groups according to the definition of DEGs. The intersection DEGs of subgroups were constituted into Venn maps (**Figures 3A–D**). We discovered a common trend among three intersecting genes across four subgroups: SLC5A5(solute carrier family5), AVPR1A(arginine vasopressin receptor 1A), and CBLN1(cerebellin 1 precursor protein). Specifically, both SLC5A5 and AVPR1A were downregulated in tumor and LNM tissues. Subsequently, we conducted survival analyses on these three genes and found that SLC5A5 and AVPR1A were associated with disease-free survival and that patients with a higher level of SLC5A5 and AVPR1A exhibited increased disease-free survival rates. However, at the same time, the higher level of SLC5A5 indicated poorer overall survival (**Figures 4A–F**).

### 3.4 GEO database of single-cell RNA sequencing

**Figure 5:**
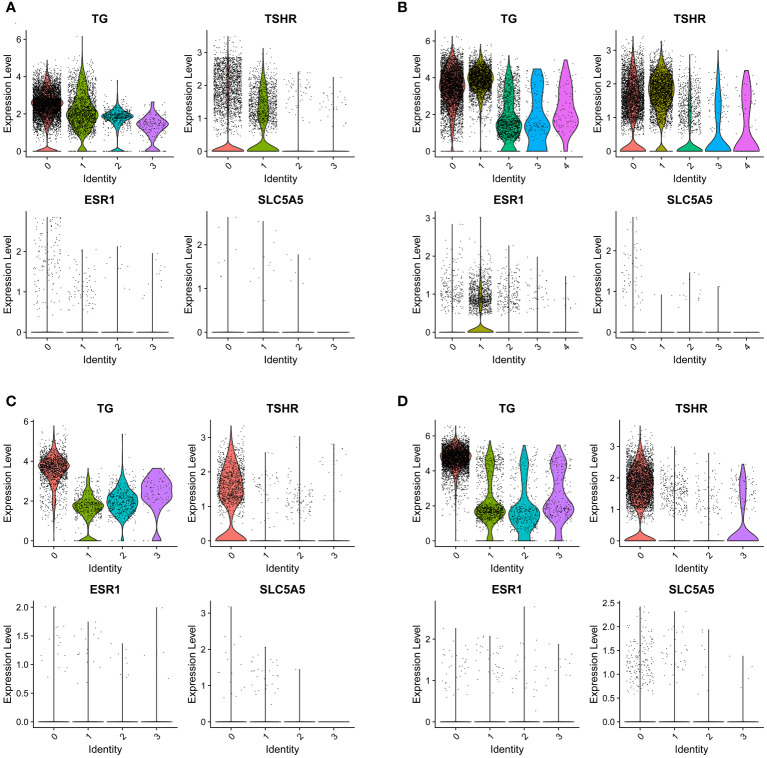
The expression of SLC5A5 and its correlation genes in four different tissues. **(A)** Tumor with lymph node metastasis; **(B)** Para-tumor tissue with lymph node metastasis **(C)** Tumor without lymph node metastasis **(D)** Para-tumor tissue without lymph node metastasis. TG, thyroglobulin; TSHR, thyrotropin receptor; SLC5A5, solute carrier family5; ESR1, estrogen receptor. The number of lateral axes represents different cell subgroups.

As members of the sodium iodide transport family, we show a strong interest in SLC5A5. By analyzing the expression of SLC5A5 across various tissues through the single-cell database, we discovered that SLC5A5 was downregulated in tumors and tissues with LNM. Furthermore, we observed a positive correlation between SLC5A5 expression and TSHR, while it appears that there is no clear association between SLC5A5 and ESR1(**Figures 5A–D**).

## 4 Discussion

LNM can serve as an indication of aggressive behavior and signs of recurrence potential in PTC (25, 26). In our study, women exhibited a greater propensity towards hypothyroidism and presented with elevated serum TSH levels, which may result from an increased likelihood of Hashimoto’s thyroiditis in women, which may contribute to the destruction of thyroid tissue during the chronic phase. It’s widely accepted that a higher TSH level promotes the proliferation of thyroid cells, leading to lymph node metastasis and invasion of surrounding tissues. Consequently, TSH suppression was theoretically an effective method to prevent the recurrence of PTC and is considered viable for low-risk PTMC patients who opt to actively monitor their condition (7). Despite some studies suggesting that TSH suppression could be beneficial for patients at high risk of recurrence, nevertheless, it was useless for low-risk patients (27). Furthermore, endogenous TSH didn’t affect the outcomes of ^131^I ablation therapy for the residual lesions (28). In our study, we found that thyroid function and the level of TSH didn’t affect the status of LNM in patients with different ages, genders, tumor size, and extrathyroidal extension. On the contrary, our research found that females had a higher level of TSH but a lower risk of LNM. For these findings, we suspected that the level of TSH wasn’t the exclusive factor that affects the aggressive behavior of PTC.

In addition to our findings, we discovered that in male and younger patients, the expression of SLC5A5 is downregulated, mirroring its expression in tissue with lymph node metastasis. As a sodium iodide transporter-related gene, the low expression of SLC5A5 was a manifestation of dedifferentiation in cells (29). Furthermore, our research indicated that a decreased expression of SLC5A5 correlated with a shorter disease-free survival time, which means male and younger patients are easier to recurrence. Individuals with a low level of SLC5A5 were less responsive to ^131^I treatment and more prone to encounter LNM and postoperative recurrences (30). In addition, our study discovered a positive correction between the expression of TSHR and ESR1 to SLC5A5, which explained why the expression of SLC5A5 is higher in females. In reality, males were more likely to be resistant to the therapy of ^131^I and experience recurrence. Consequently, we propose that patients should receive individualized doses of ^131^I, with men requiring a higher dose. As a sodium iodide transporter-related gene, SLC5A5 was regulated by upstream gene mutations and epigenetic changes, therefore drugs targeting SLC5A5 can be studied for use in ^131^I-resistant patients (31–33).

As a conclusion of our single-center study, it becomes imperative to collaborate with other centers to verify the reliability of the results. Additionally, our study merely captured the preoperative examinations; hence, we want to aim to accumulate more detailed insights into the postoperative follow-up to dynamically assess the intricacies of the treatment process. Lastly, we require additional evidence based on the molecular evidence to reinforce our conclusions.

## 5 Summary

Regarding the uncertain influence of TSH on the behavior of PTC, a flexible approach to TSH suppression indications for patients at risk of cardiovascular disease or those experiencing menopausal women was needed. Additionally, variations in ^131^I absorption capabilities due to SLC5A5 expression levels across different genders and ages suggest that postoperative PTC therapies should take into account these demographic differences to minimize patient harm.

## Data availability statement

The datasets presented in this study can be found in online repositories. The names of the repository/repositories and accession number(s) can be found in the article/**Supplementary Material**.

## Ethics statement

The studies involving humans were approved by The Ethics Committee of the Second Hospital of Tianjin Medical University. The studies were conducted in accordance with the local legislation and institutional requirements. Written informed consent for participation was not required from the participants or the participants’ legal guardians/next of kin in accordance with the national legislation and institutional requirements.

## Author contributions

CY: Conceptualization, Data curation, Formal analysis, Investigation, Methodology, Software, Writing – original draft, Writing – review & editing. J-JS: Conceptualization, Funding acquisition, Project administration, Resources, Supervision, Visualization, Writing – review & editing. XH: Conceptualization, Funding acquisition, Project administration, Resources, Visualization, Writing – review & editing. LJ: Data curation, Formal analysis, Methodology, Software, Supervision, Writing – review & editing.
